# Application of BERT to Enable Gene Classification Based on Clinical Evidence

**DOI:** 10.1155/2020/5491963

**Published:** 2020-10-07

**Authors:** Yuhan Su, Hongxin Xiang, Haotian Xie, Yong Yu, Shiyan Dong, Zhaogang Yang, Na Zhao

**Affiliations:** ^1^National Pilot School of Software, Yunnan University, Kunming, 650091, China; ^2^Department of Mathematics, The Ohio State University, Columbus, OH 43210, USA; ^3^Department of Radiation Oncology, University of Texas Southwestern Medical Center, Dallas, TX 75390, USA

## Abstract

The identification of profiled cancer-related genes plays an essential role in cancer diagnosis and treatment. Based on literature research, the classification of genetic mutations continues to be done manually nowadays. Manual classification of genetic mutations is pathologist-dependent, subjective, and time-consuming. To improve the accuracy of clinical interpretation, scientists have proposed computational-based approaches for automatic analysis of mutations with the advent of next-generation sequencing technologies. Nevertheless, some challenges, such as multiple classifications, the complexity of texts, redundant descriptions, and inconsistent interpretation, have limited the development of algorithms. To overcome these difficulties, we have adapted a deep learning method named Bidirectional Encoder Representations from Transformers (BERT) to classify genetic mutations based on text evidence from an annotated database. During the training, three challenging features such as the extreme length of texts, biased data presentation, and high repeatability were addressed. Finally, the BERT+abstract demonstrates satisfactory results with 0.80 logarithmic loss, 0.6837 recall, and 0.705 *F*-measure. It is feasible for BERT to classify the genomic mutation text within literature-based datasets. Consequently, BERT is a practical tool for facilitating and significantly speeding up cancer research towards tumor progression, diagnosis, and the design of more precise and effective treatments.

## 1. Introduction

Nowadays, genomic, transcriptomic, and epigenomic studies have been benefited from the development of inexpensive next-generation sequencing technologies, which play essential roles in exploring tumor biology [1–3]. Tumors usually possess heterogeneities, and the genomic profiling of tumors normally contains various types of genetic mutations [4–7]. However, only a small proportion of mutation genes are involved in boosting tumor growth, whereas most of them are neutral and irrelevant to tumor progression [8, 9]. Characterization and identification of cancer driver genes are important a in clinical trials to reveal tumor pathogenesis and facilitate diagnosis, prognosis, and personalized therapy [10–13]. Despite the importance of gene classification, the following analysis is challenging due to the significant amount of manual work for interpretating genomics, which is time-consuming, laborious, and subjective. With the increasing availability of electronic unstructured and semistructured data sources, automatically categorizing documents has emerged as a potential tool for information organization. Machine learning (ML), as a promising optimization tool, has been widely used in credit scoring, fraud detection, retailers, market segmentation, manufacturing, education, and healthcare [14–18]. Hence, using ML to analyze clinical contextual data automatically is favorable [19–21]. For example, in 1986, Swanson first discovered the undiscovered links in a large number of scientific literature [22]. Also, Marcotte et al. used Naive Bayesian classification to classify the literature focusing on protein-protein interaction [23].

Despite the achievements traditional ML methods have made, potential drawbacks such as low accuracy exist when they are applied on clinical text classification. In 2018, Google proposed that the BERT method achieved state-of-the-art results in 11 projects, including text classification [24]. Descriptions about clinical research acadamic papers show high similarities , which blurs the classification boundary, increases the inconsistancy, and lows the accuracy. Consequently, the advanced ML methods, such as Light Gradient Boosting Machine (LightGBM), has been proposed to enable gene multiclassification based on complex literature [25]. Nevertheless, these methods are limited by complex calculations when applied to large-scale datasets, particularly for genomic-related literature datasets that contain millions, or billions, of annotated training examples [26, 27]. In addition, the performances of ML are dependent on feature extraction that requires professional knowledge and long-term processing [28–31].

To overcome these difficulties, deep learning (DL) has emerged to handle large-scale and complex datasets since its performance increases with the enlargement of datasets [32–34]. For example, the convolutional neural networks (CNN) [35], recurrent neural networks (RNN) [36], and their combination [37] have been applied to the sentence classification successfully. Also, In 2018, Google proposed that the BERT method achieved state-of-the-art results in 11 projects, including text classification.

Hence, we fine-tune the BERT model to classify mutation effects (9 classes) using an expert-annotated oncology knowledge base. Our BERT method is developed based on the original BERT model and is capable of obtaining different syntactic and semantic information. Three main characters of training datasets including extreme length of text entry, data imbalance, and repetitive description are engineered during training challenges. We propose three truncation methods including abstract+head, head only, and head+tail to deal with extreme length of text entry and repetitive description. Besides, data imbalance is relieved by negative sampling. Overall, we improve the BERT method to classify complex clinical texts, and obtain 0.8074 logarithmic loss, 0.6837 recall, and 0.705 *F*-measure scores.

## 2. Problem Statement

The treatment of cancer is closely related to the identification of mutant genes [38]. At present, clinicians need review and classify each mutant gene manually according to the evidence in text-based clinical literature, which is a complicated, time-consuming, and error-prone method [39–42]. To solve this problem, Memorial Sloan Kettering Cancer Center (MSKCC) has provided an expert-annotated precision oncology knowledge base with thousands of mutations manually annotated by world-class researchers and oncologists for studying gene classification using computer-based method [43]. On top of that, we design an artificial intelligence algorithm to automatically and accurately classify mutations for avoiding mistakes caused by manual classification, and provide further help for cancer treatments.

In recent years, with the rise of artificial intelligence, natural language processing, which uses linguistics, computers, mathematics, and other scientific methods to communicate between human beings and computers, has developed rapidly [44–46]. Among them, text classification is one of the most basic and critical tasks in natural language processing [47]. Text classification is the process of associating a given text within one or more categories according to characteristics of texts (content or attributes) under a predefined classification system [48–50]. The process of text classification mainly includes three steps. Firstly, the text is preprocessed, then the vector representation of the text is extracted. Finally, the classifier is trained to classify the text [48]. Text classification can be divided into single-label text classification and multilabel text classification according to the number of labels to which the text belongs. The single-label text refers to each text belonging to only one category, while multilabel text refers to each text belonging to one or more categories [51–53]. The calculation formula for text classification can be defined as follows:

(1) $$\begin{array}{c} F\left(D,C\right)=\left\{\text{True},\text{False}\right\}. \end{array}$$

In the formula, the collection *D* = {*d*_1_, *d*_2_, ⋯*d*_*n*_} refers to the set of texts classified, where the *i*th classified text is represented by d_i_, and *n* is the number of classified texts. The collection *C* = {*c*_1_, *c*_2_, ⋯, *c*_*m*_} is a collection of predefined classification categories, where the *j*th category is represented by *c*_*j*_, and *m* is the number of predefined categories. *F* is a function representing a mapping relationship.

Currently, the most common methods for text classification are statistical ML and DL-based methods. Statistical ML methods usually preprocess texts in the first place, then manually extract high-dimensional sparse features. Consequently, they use statistical ML algorithms to obtain classification results. In 1998, Joachims first employed support for vector machine (SVM) in text classification and achieved favorable results [54]. In the following research, many methods based on statistical ML are used in text classification, including Naïve Bayes classifier [55], *K*-nearest Neighbor method (KNN) [56], decision tree [57], boosting [58], and LightGBM [59]. Among them, LightGBM is widely used in classification problems due to its fast speed, low memory consumption, and relatively high accuracy [60]. Although LightGBM gets good classification results in some scenes, research related to this approach runs basically into bottleneck due to its strong dependence on the effectiveness of features. Also, it is time-consuming and labor-intensive during feature extraction process.

Although the traditional statistical ML models can classify texts faster than the manual method, they require manual feature extraction, which leads to a large amount of labor cost and is difficult to obtain effective features [61–63]. On the other hand, the DL methods are superior to traditional statistical ML methods in terms of text feature expression and automatic acquisition of feature expression capabilities, thus eliminating complex manual feature engineering processes and reducing possible application costs [64]. As we all know, large-scale pretraining language models have become a new driving force for various natural language processing tasks [65]. For example, BERT models can significantly improve model performance by fine-tuning downstream tasks. Google first proposed the BERT model, and it completely subverted the logic of training word vectors before training specific tasks in natural language processing [24]. Methods of fine-tuning the BERT model, such as extended text preprocessing and layer adjustment, have been proved to improve the results substantially [66]. Wu et al. proposed a conditional BERT method, which can enhance the text classification ability of original BERT method by predicting the conditions of masked words [67]. To sum up, it is feasible to employ the fine-tuned model based on the original BERT to classify genetic mutations.

Hence, we propose an improved BERT model with high classification accuracy after analyzing the MSKCC mutation gene interpretation database thoroughly. We believe this method can be successfully applied to genetic mutation classification. The main contributions of our work are summarized as follows:

1. The text description of the individual sample shows considered lengths. There are differences in text lengths between different categories of samples. Some categories contain shorter words, while others contain miscellaneous descriptions. Generally, texts in a dataset range from hundreds to thousands of words in length. However, the lengths of the gene mutation in this paper are much longer than usual. We use the BERT method to truncate texts and extract valuable information in the texts using different methods, thus avoiding adverse impacts of excessive differences in text lengths on the results.
2. There is a deviation of total gene number in all categories. Individual genes are unevenly distributed in different categories. Some genes belong to five or more groups, while others only present in two categories. To solve the vast differences in the number of samples between different categories in the dataset, we choose an undersampled data processing method to balance the data deviations between different categories.
3. The whole dataset has a high repeated description. Different examples belong to different categories share the same text entry. Some categories show a high correlation, which may lead to low accuracy. To solve this problem, we improve the BERT model and splice the last three layers of the initial model, which increases the accuracy of the model and reduces the running time.
4. To a certain extent, we illustrate the effectiveness of using DL in the classification of genetic clinical texts. As the data set increases, the DL model represented by BERT will learn the characteristics of the sample better to achieve exceptional results. In the future, DL models will have better performances on similar tasks.

## 3. Materials and Methods

### 3.1. Description of Datasets

MSKCC sponsored the training and test datasets in this study for method development and validation. For the past several years, world-class experts have created a clinical evidence annotated precision oncology knowledge database. The annotations contain information about which genes are oncology clinically actionable. We sum up three characteristics of the MSKCC datasets mentioned below:

- Textual descriptions of individual samples exhibit considerable lengths. The text lengths among different classes show variabilities. Some of the classes contain shorter words while other classes contain redundant descriptions.
- The overall gene numbers presented among the whole classes show biases. The distribution of individual genes in different classes is unequal. Some genes belong to five classes or more, and some of the genes only fit in two classes.
- High repetitive descriptions exist in the whole datasets. Different samples belong to different classes that share the same text entry. Classes demonstrate high correlations.

#### 3.1.1. Length of Entry Text

**Figure 1:**
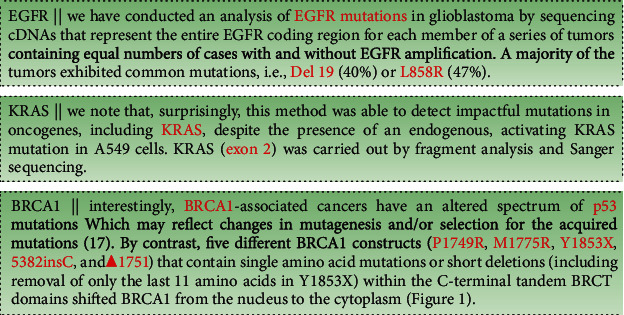
The cut-off document views of the datasets.

**Figure 2:**
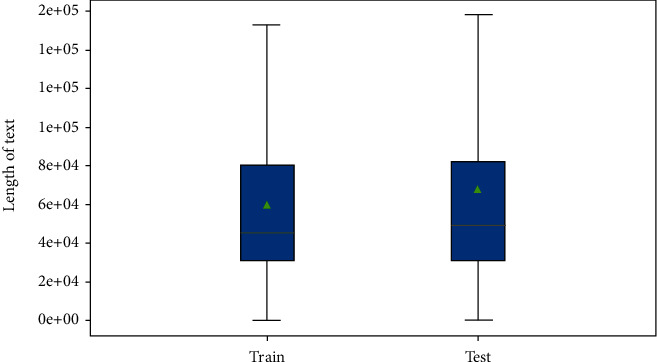
Distribution of the text entry lengths.

**Figure 3:**
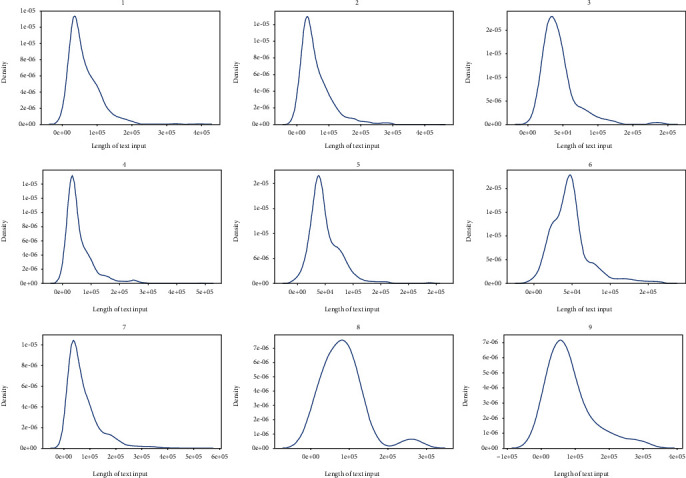
Distribution of the text entry lengths among different classes.

It is reasonable to analyze the length of the entry text as a prior task for textual-based classification. We find that extremely long descriptions with massive irrelevant information are correlated with samples (Figure 1). We plot the distribution of text lengths (Figure 2), and our datasets contain more counted words than the normal classification datasets in reviews [68]. Consequently, we examine the distribution of text lengths among different target classes to better understand the uniformity of datasets. Variabilities are demonstrated among different classes (Figure 3). Comparing the density of the length distributions, we divide the classes into three groups. Classes 3, 5, and 6 contain the shortest counted words; classes 1, 2, 4, and 7 exhibite medium counted words; and classes 8 and 9 show the most counts. Overall, two features that increase the task difficulty are attracted: considerable lengths of words and the unequal text length distribution among different classes.

#### 3.1.2. Analysis of the Data Distribution

**Figure 4:**
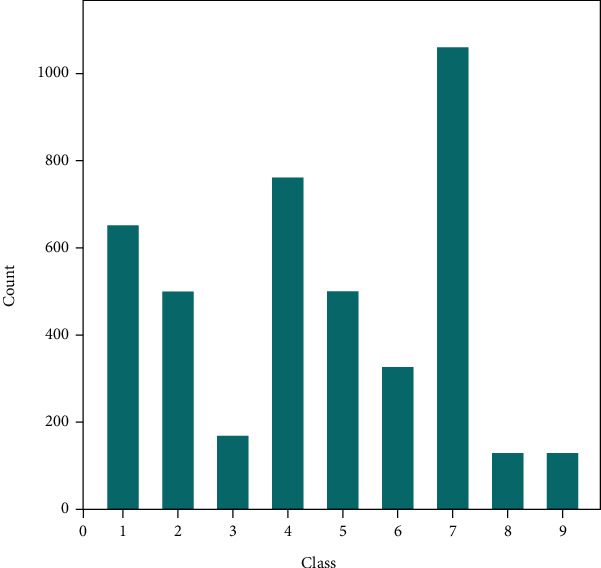
Distribution of the number of genes among 9 classes.

Analyzing the composition of datasets can help us construct algorithms at an early stage. We sum up the frequency of genes among 9 classes (Figure 4). The 9 classes correspond to mutation effects but are annotated using numbers instead of real textural information to avoid artificial labeling, thus improving the reliability of our algorithms during the training. The true information of these labels is listed in Table 1. The distribution of genes among 9 classes exhibited bias. Genes in class 7 are significantly higher than genes in classes 3, 8, and 9.

**Figure 5:**
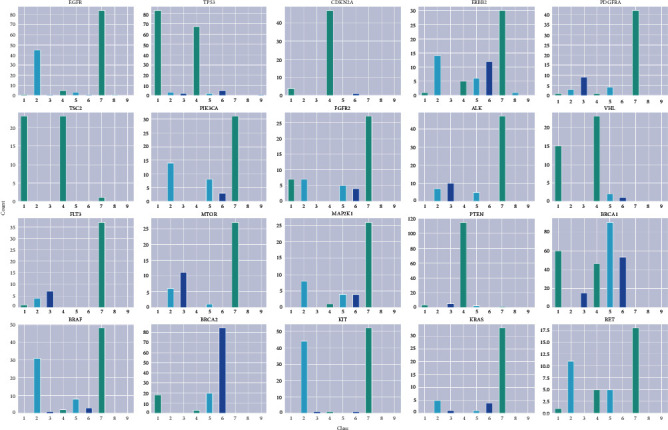
Distribution of genes among classes.

We also examin the interactions among different features within target classes. To reduce calculations, we select the top 20 gene types to illustrate the interrelations instead of the whole gene types (Table 2). Selected genes are sorted by classes (Figure 5). The distribution of genes demonstrate huge variabilities among different classes. We find that classes 8 and 9 contain almost none of these genes, and class 3 contain a few of these genes. These distribution biases are in accordance with our previous gene frequency summary based on the whole gene types. Similarly, the trends in classes 1, 2, 4, and 7 correspond to our previous results. These comparable results indicate that the whole datasets are highly associated with selected genes. Consequently, discriminatory differences among classes can impede the feature learning performances of our algorithms and low the accuracy of the text classification.

We further explore the distribution of individual genes within classes, which demonstrates inequitable distributions. For instance, genes such as CDKN2A, PTEN, and TSC2 only present in a limited number of classes (lesser than three). In contrast, BRCA1, ERBB2, FGFR2, and RET are possessed in the majority of classes. Compared with genes only present in a few groups, genes that spread among classes are generally difficult to classify because elaborate texture descriptions can blur the classification standard. Hence, the accuracy of classifications is dependent on the gene compositions. Commonly, genes distributed in lesser classes can show more satisfactory results.

#### 3.1.3. Characteristics of the Datasets

Using typical genes as samples, we find that these typical genes presented in classes demonstrated variabilities. To better recognize these biases and complete potential influences behind them, we conduct a statistical analysis of the whole datasets from the text entry aspects. We find that different samples share the same text entries after extracting common words. The highly repetitive descriptions increase the difficulties of classification, especially when samples in different classes share the same sketches. The worst scenario is the fact that samples belong to different classes that have the same name, but other clue information is missing. For example, five possible mutations of gene BRCA1, the mutation P1749R, M1775R, Y1853X, 5382insC, and *Δ*1751, may belong to different classes, but their descriptions are close, even in the same sentence. Similarly, two mutations of EGFR, such as Del 19 and L858R, also show in pairs (Figure 1). Hence, we can assume that it is tough to categorize the samples into correct classes by relying on the name of mutations with limited or without other valuable information.

**Figure 6:**
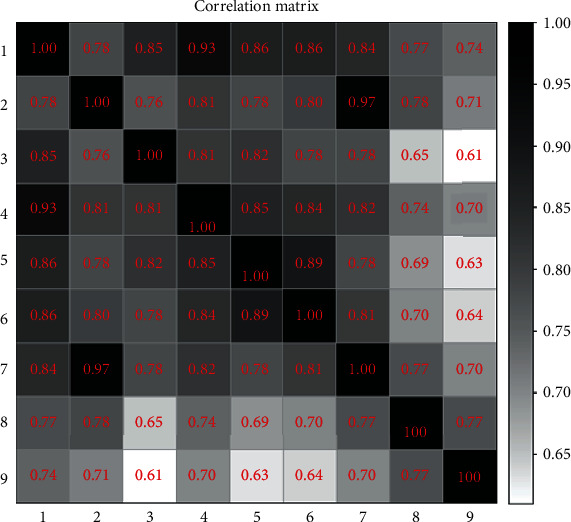
Confusion matrix analysis of the similarity of the texts in different classes.

Also, class-dependent word similarities are evaluated using full word lists (Figure 6). Correlation coefficients exhibited high connections (higher than 60%) between classes. Among them, classes 2 and 7 and classes 1 and 4 demonstrate extremely high correlations with 97% and 93% coefficients, respectively. Therefore, we think substantial work needs to be done to clarify samples that share similar descriptions in high correlative classes. Besides, we can not expect high accuracy when classifying samples with these properties.

### 3.2. BERT

Compared with traditional ML methods, DL demonstrates better performances in text feature expression and automatically obtains feature expression capabilities, thus removing the complicated manual feature engineering process and decreasing its application cost. BERT is a new language representation model based on DL, which was released by the AI team of Google company in October 2018. The BERT model is divided into two parts: pretraining and fine-tuning.

#### 3.2.1. Pretraining of Modified BERT Model

In the pretraining process, a large-scale unlabeled text corpus is used to complete the deep vector representation of text content in the deep bidirectional neural network through an unsupervised training method, thus forming the corresponding text pretraining model. Google has trained two pretrained models. One is the BERT-base model, which includes 12 transformers, 12 self-attention heads, and 768 hidden sizes. The other is the BERT-large model, which contains 24 transformers, 16 self-attention heads, and 1024 hidden sizes. Parameters of BERT-base methods are loaded into the downstream BERT classification model so that our model parameters can be fine-tuned based on these pretrained models, which significantly reduces the convergence time of the model and increases the accuracy of the model. During the pretraining process, BERT randomly masks out, replaces some words, and predicts these missing or replaced words through the remaining ones. The transformer must maintain a distributed representation of each input token. The transformer is likely to remember the word masked without this masking and predicting procedure.

#### 3.2.2. Fine-Tuning of Modified BERT Model

Since the generalization ability of the pretrained model is powerful, the BERT pretrained model can be applied to various downstream tasks after fine-tuning the parameters of the pretrained model. For example, it is possible to meet the needs of a text classification task by adding pooling, full connect, and Softmax function to the output layer sequence of fine-tuned BERT model. The fine-tuning process requires much lesser training resources compared to the pretraining process. The method of fine-tuning BERT model, such as truncation and layer adjustment, has been proved to be capable of improving the result [18]. It implements the process of unsupervised learning through the mask, thereby predicting the vocabulary that will appear in the sentence and understanding the specific meaning of the sentence according to the context.

### 3.3. Evaluation Equation

This paper evaluates the performances of the model using several evaluation indicators: Logloss, recall (REC), precision (PRE), F1 score, receiver operating characteristic (ROC) curve, and confusion matrix. True Positive (TP), True Negative (TN), False Positive (FP), and False Negative (FN) can be used to calculate some of the indicators mentioned above. TP is the number of categories that are correctly predicted. TN is the number of categories that are correctly predicted as another class. FP is the number of categories that are wrongly predicted. FN is the number of categories that are wrongly predicted as another class.

In multiclassification tasks, Logloss is one of the most common loss functions, where the predicted input is a probability value distribution between 0 and 1, and it can be defined as follows:

(2) $$\begin{array}{c} \text{Logloss}=-\frac{1}{S_{n}}\overset{S_{n}}{\underset{m=1}{\sum }}\overset{N}{\underset{n=1}{\sum }}y_{mn}\log\left(p\left(y_{mn}\right)\right), \end{array}$$

where *M* is the number of samples and *N* is the number of classifications. *y*_*mn*_ is the predicted result of classification, such as 0 and 1. *p* (*y*_*mn*_) is the predicted probability of *y*_*mn*_.

PRE defines the proportion of genes identified correctly belonging to this type of mutation:

(3) $$\begin{array}{c} \text{PRE}=\frac{\text{TP}}{\text{TP}+\text{FP}}. \end{array}$$

REC calculates the proportion of genes identified correctly belonging to this type of mutation in all this type of gene:

(4) $$\begin{array}{c} \text{REC}=\frac{\text{TP}}{\text{TP}+\text{FN}}. \end{array}$$

F1 score takes into account the factors of PRE and REC. F1 is the standard metric for this task. It combines precision and recall. Macro-F1 is a parameter index that can best reflect the effectiveness and stability of the model:

(5) $$\begin{array}{c} \text{F}1=\frac{2\text{PRE}*\text{REC}}{\text{PRE}+\text{REC}}. \end{array}$$

The ROC curve is created by plotting the TP against the FP at various threshold settings.

The confusion matrix is a specific table capable of visualizing the performance of an algorithm. Individual rows of the matrix represent the predicted gene classses, while each column represents the genes in the actual classes.

## 4. Experiments

For easier comparison with other methods, our training process uses the GPU of the server in the lab for training. There are 3136 training sets and 553 verification sets in total. The Python language is selected as the programming language in this experiment. The experiment is completed on Tensorflow's open-source framework and BERT-base. We use the parameters on BERT-base trained by Google through a large number of corpus on Wikipedia as pretraining parameters to accelerate the convergence speed and reduce the convergence difficulty. Our experimental parameters are batch size 128, learning rate 3*e* − 5, and warmup period 0.06; the whole experiment runs for 30 cycles; the maximum sequence length of BERT input is 512; and the optimizer is Adam optimizer, while other model parameters remain unchanged.

### 4.1. Experiment Procedure

The BERT model can automatically complete the process of converting each word in the text into a one-dimensional vector by querying the word vector table and inputting it in the model. The input of the model contains three sections: the token embeddings, the segmentation embeddings, and position embeddings.

**Figure 7:**
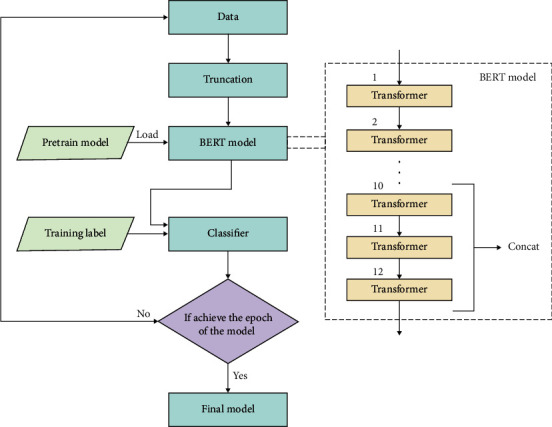
Scheme of the training.

Because BERT is a pretraining model with high generalization ability, the output layer of BERT can be externally connected with corresponding layers to complete downstream tasks. For example, in this experiment, the processed data is substituted into the BERT model for training, and the output layer will connect Softmax function for classification tasks (Figure 7).

BERT is an unsupervised model that uses whether the sentences are related to each other as labels and masks some words to make the masked words as labels, thus avoiding the tedious process of manually labeling data. Generally, the data in the dataset are not balanced. Take the samples in the 7th and 8th categories of the dataset as an example. The difference between their numbers even reaches more than 10 times. In this case, the default classification method makes classifiers pay too much attention to the category with a larger number of samples, thus making the generalization ability of the model weak and unable to obtain satisfactory results. Therefore, we use random sampling to eliminate the imbalance between data and extract only a part of samples from the category within a larger number of samples to balance the sample number differences between classes.

Simultaneously, because the length of the gene text in the dataset is greater than 512 tokens, which is the longest length that can be retained by BERT, we need to use the truncation method to intercept part of the information in the text. We take three ways to solve this problem. The head only truncation method intercepts the first 512 tokens (at most) as input, the head+tail method intercepts part of the head and part of the tail to form 512 tokens (at most) as input, and the abstract+head method sorts the gene text according to importance, then select the most important 512 tokens (at most) as input.

Finally, the processed data are substituted into the BERT model for training. Numerous previous works have shown that fine-tuning a pretrained model which has been trained with a large amount of corpus can significantly improve the classification result. As BERT can learn different contents in different layers, stitching some of the layers together can make the model get richer information, thereby improving the accuracy of the model, so the last three layers in the BERT model are concatenated. Max pooling, fully connected, and Softmax function are added after the concatenated output layer to realize the classification of gene text to improve the classification accuracy of the model.

### 4.2. Experiment Results and Discussions

It can be seen from the figure that compared with the LightGBM method, the BERT methods using three types of truncation have higher ACC, REC, and F1 score. The confusion matrix shows our classification situation in a visual way (Figure 6). The red numbers are nonzero values. It can be observed that type 1 is easy to be confused with types 4 and 5. There are more machine judgment errors of texts between type 7 and type 2. Overall, the classification of data-lacking types 8 and 9 is more complicated than other types, possibly because there are fewer samples of types 8 and 9, and these two types have fewer intersections with other types of mutation. The lack of intersection leads to difficulties in distinguishing types 8 and 9 from different types of mutations. The ROC curve can evaluate the accuracy of the model prediction.

**Figure 8:**
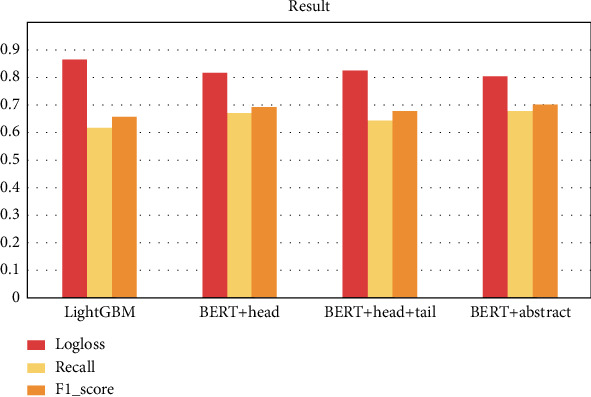
Evaluation of four methods.

The performance and ranking of the entries for the proposed four methods are shown in Figure 8. All methods share the same setting of hyperparameters for an unbiased comparison. Overall, deep learning-based algorithms (BERT) perform slightly better than machine learning-based methods (LightGBM). Among the three models using BERT, the BERT+abstract truncation method has the best performance as a single model with 0.8074 logarithmic loss and 0.6837 recall. The 0.705 *F*-measure score is limited by the extreme shortage of training data. Better performance should be obtained when it is applied to large-scale datasets.

**Figure 9:**
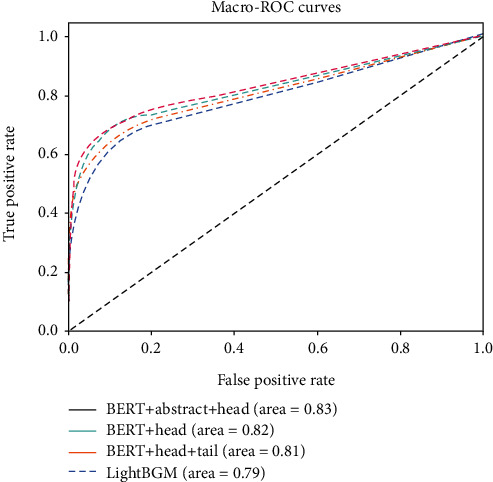
ROC curves of the proposed methods.

Besides, the ROC curves of the other three methods are below the ROC curve of the BERT+abstract (Figure 9). The ROC curves for the BERT+abstract, the BERT+head, the BERT+head+tail, and the LightGBM with the highest and lowest AUCs are also shown in Figure 9. Compared results indicate better performance of the BERT+abstract since the AUC assesses the algorithm's inherent validity using an effective and combined measure of sensitivity and specificity. The accuracy of predicted results is highly dependent on the datasets. The performances of our model are limited by the size of available datasets in our case. However, the capabilities of deep networks can be improved using expanded data. Our proposed model is a proof-of-concept, and we believe it is applicable when applied on large-scale datasets.

**Figure 10:**
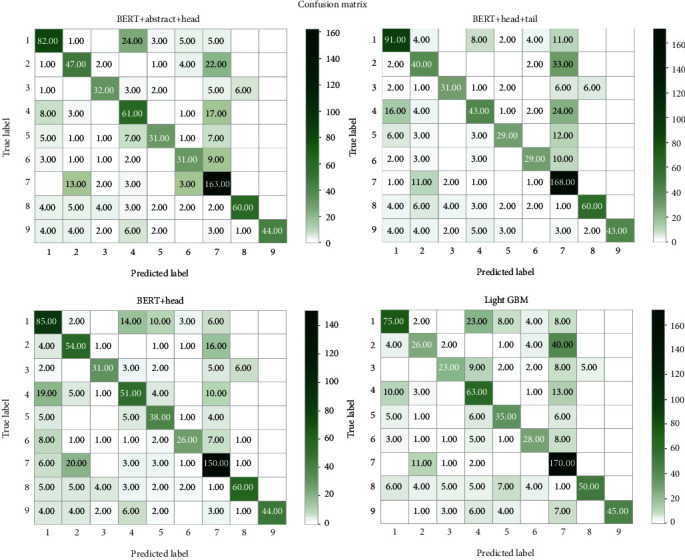
Confusion matrix tables of proposed four methods.

Moreover, we compare confusion matrix tables using predict classes versus true classes among different methods (Figure 10). The confusion matrix table is an error matrix which can be used to evaluate the performance of the algorithm. In summary, individual classes of genes are predicted precisely using the BERT+abstract method, corresponding with results of Logloss and F1 measurements.

It can be observed that class 1 is easy to be confused with classes 4 and 5. Furthermore, there are more machine judgment errors of texts between type 7 and type 2. These phenomena can be easily attributed to the similarity of texts among these classes as we previously described. Also, it is apparent that classifying classes 8 and 9 is complicated. The computer may misjudge mutation texts with real labels of 8 or 9 as other types but hardly underestimate other types of mutation texts as type 8 or 9 since there are fewer samples in classes 8 and 9. The shortage of samples in classes 8 and 9 also fails to provide sufficient data to distinguish themselves from other classes since there are no intersections. Contrastingly, the classification of class 7 is easier due to the abundant samples. Therefore, the abundance of data plays essential roles in improving the efficiency of classification.

## 5. Conclusion

In this study, we propose a deep learning algorithm to identify genomic information within texture-based literature abstracts. Aiming to address the classification problem in an extremely long, imbalanced, and repetitive dataset, we test four methods, including LightGBM and three different truncation BERT methods. By analyzing their Logloss, recall, F1 score, ROC curve, and AUC scores, we notice that the abstract+head truncation BERT method has superior results than other algorithms in all indicators.

In this study, our BERT method is limited due to the shortage of datasets, and its performance can be improved dramatically with the size of datasets increasing. Moreover, our approach will be potentially applied on diagnosing and treating more than 120,000 patients every year around the world based on the announcement of the MSKCC, which will provide our opportunity to enhance our methods further when large-scale datasets are available. We believe BERT is a promising tool for accelerating tumor genomic-related research and facilitating tumor diagnosis and treatments. Besides, this text-based classifier algorithm demonstrated high universality, and it is applicable not only in tumor-specific research but also in other types of diseases and in other nonacademic areas.

## Figures and Tables

**Table 1:** Class information corresponds to the annotated number.

Annotated number	Class information
1	Likely loss of function
2	Likely gain of function
3	Neutral
4	Loss of function
5	Likely neutral
6	Inconclusive
7	Gain of function
8	Likely switch of function
9	Switch of function

**Table 2:** List of top 20 genes in the datasets.

Rank	Gene name	Rank	Gene name
1	EGFR	11	FLT3
2	TP53	12	MTOR
3	CDKN2A	13	MAP2K1
4	ERBB2	14	PTEN
5	PDGFRA	15	BRCA1
6	TSC2	16	BRAF
7	PIK3CA	17	BRCA2
8	FGFR2	18	KIT
9	ALK	19	KRAS
10	VHL	20	RET

## Data Availability

All data generated or analyzed during this study are included in this published article.
